# Traceable Research Data Sharing in a German Medical Data Integration Center With FAIR (Findability, Accessibility, Interoperability, and Reusability)-Geared Provenance Implementation: Proof-of-Concept Study

**DOI:** 10.2196/50027

**Published:** 2023-12-07

**Authors:** Kerstin Gierend, Dagmar Waltemath, Thomas Ganslandt, Fabian Siegel

**Affiliations:** 1 Department of Biomedical Informatics at the Center for Preventive Medicine and Digital Health Medical Faculty Mannheim, Heidelberg University Mannheim Germany; 2 Core Unit Data Integration Center and Medical Informatics Laboratory University Medicine Greifswald Greifswald Germany; 3 Chair of Medical Informatics Friedrich-Alexander-Universität Erlangen-Nürnberg Erlangen Germany

**Keywords:** provenance, traceability, data management, metadata, data integrity, data integration center, medical informatics

## Abstract

**Background:**

Secondary investigations into digital health records, including electronic patient data from German medical data integration centers (DICs), pave the way for enhanced future patient care. However, only limited information is captured regarding the integrity, traceability, and quality of the (sensitive) data elements. This lack of detail diminishes trust in the validity of the collected data. From a technical standpoint, adhering to the widely accepted FAIR (Findability, Accessibility, Interoperability, and Reusability) principles for data stewardship necessitates enriching data with provenance-related metadata. Provenance offers insights into the readiness for the reuse of a data element and serves as a supplier of data governance.

**Objective:**

The primary goal of this study is to augment the reusability of clinical routine data within a medical DIC for secondary utilization in clinical research. Our aim is to establish provenance traces that underpin the status of data integrity, reliability, and consequently, trust in electronic health records, thereby enhancing the accountability of the medical DIC. We present the implementation of a proof-of-concept provenance library integrating international standards as an initial step.

**Methods:**

We adhered to a customized road map for a provenance framework, and examined the data integration steps across the ETL (extract, transform, and load) phases. Following a maturity model, we derived requirements for a provenance library. Using this research approach, we formulated a provenance model with associated metadata and implemented a proof-of-concept provenance class. Furthermore, we seamlessly incorporated the internationally recognized Word Wide Web Consortium (W3C) provenance standard, aligned the resultant provenance records with the interoperable health care standard Fast Healthcare Interoperability Resources, and presented them in various representation formats. Ultimately, we conducted a thorough assessment of provenance trace measurements.

**Results:**

This study marks the inaugural implementation of integrated provenance traces at the data element level within a German medical DIC. We devised and executed a practical method that synergizes the robustness of quality- and health standard–guided (meta)data management practices. Our measurements indicate commendable pipeline execution times, attaining notable levels of accuracy and reliability in processing clinical routine data, thereby ensuring accountability in the medical DIC. These findings should inspire the development of additional tools aimed at providing evidence-based and reliable electronic health record services for secondary use.

**Conclusions:**

The research method outlined for the proof-of-concept provenance class has been crafted to promote effective and reliable core data management practices. It aims to enhance biomedical data by imbuing it with meaningful provenance, thereby bolstering the benefits for both research and society. Additionally, it facilitates the streamlined reuse of biomedical data. As a result, the system mitigates risks, as data analysis without knowledge of the origin and quality of all data elements is rendered futile. While the approach was initially developed for the medical DIC use case, these principles can be universally applied throughout the scientific domain.

## Introduction

Provenance—a piece of metadata—is considered information that is fundamental in the data life cycle because it expresses the traceability of the processed data and facilitates the reproducibility of the results [1,2]. The availability of provenance throughout the data life cycle is deemed a crucial factor for maintaining trust in the data at all stages [3]. The data life cycle encompasses data generation, processing, validation, analysis, reporting, and application for decision-making in any context, culminating in storage within a specified retention period [4]. Medical data integration centers (DICs), particularly those established within the German Medical Informatics Initiative, must enhance accountability for their activities. This is particularly crucial for the methods used in extracting, transforming, and loading sensitive patient data from heterogeneous clinical routine systems into (standardized) research data repositories for subsequent secondary use [5]. In this given context, it is necessary to understand the limitations of the provided data [6]. Collecting comprehensive and pertinent contextual provenance information along these processing pipelines is one approach to enhance the accountability of the medical DIC (Textbox 1). Provenance and integrity must be systematically evaluated and documented in routinely collected data sets to facilitate their reuse in clinical trials [7].

**Accountability in a German medical data integration center.**

Accountability means accepting responsibility for activities and in this context entails all procedures and processes for data managing pipelines [8]. This includes keeping the movement of data elements transparent and traceable. Provenance traces enable documentation of this movement and hence generate trust in the data integrity and reliability of the provided data for secondary use.

To achieve reproducibility [9] and integrity when exchanging data between academia and industry, researchers must adhere to essential research principles, particularly following good practice guidelines (eg, good clinical practice, good research/scientific practice, commonly referred to as GxP) [10]. Ensuring and evaluating data integrity and data provenance are anticipated to be prerequisites for clinical trial data [11]. For instance, the clinical research data quality standard ALCOA+ (Attributable, Legible, Contemporaneous, Original, and Accurate+) articulates enhanced data integrity properties and fundamentally contributes to provenance information [12]. These properties pertain to attributable, legible, contemporaneous, original, accurate, complete, consistent, enduring, and available data characteristics [10].

In addition to adhering to good scientific practice [13], heightened legal requirements such as compliance with the General Data Protection Regulation (GDPR) in the European Union, or contractual obligations, mandate evidence-based data processing for both deidentification and reidentification of data, encompassing the life cycle of the patient’s consent [14].

A crucial factor in advancing these objectives is the metadata acquired from the data transformation and integration process throughout the data life cycle. The field of biological research has already acknowledged the significance of metadata, as outlined in ISO norms such as ISO/CD 20961 [15] and ISO/TC 276/WG5 on data processing and integration [16]. ISO 20961, for example, specifies requirements for the consistent formatting and documentation of data and metadata.

Furthermore, the FAIR (Findability, Accessibility, Interoperability, and Reusability) guiding principles for data management and data stewardship emphasize the overall relevance of metadata for the data itself, including those used in infrastructures and services [17]. Aspects of the FAIR recommendations explicitly address provenance capture. As such, the “R1.2” FAIR principle demands machine-accessible and readable metadata, which include provenance information about the data creation or generation. Related metadata accumulate not only during the data transformation itself but also within the software used [18]. The principle “R1.3” expects metadata to be adhering to domain-relevant community standards such as the HL7 Fast Healthcare Interoperability Resources (FHIR) or Dublin core [1]. FHIR is an internationally recognized standard that supports the exchange of data between different software systems within the health care sector [19]. In this vein, the FHIR resource “provenance” records entities and processes involved in creating a specific resource. From a technical point of view, the FHIR Provenance resource is founded on the framework of the open W3C standard PROV-Data Model definition and ontology [20], the successor to the Open Provenance Model [21]. Here, the concepts of linked entities, activities, and agent resources enable the establishment of a provenance model. Such resources can be described with the W3C Resource Description Framework (RDF) method [22]. RDF is a data model, which is commonly stored in formats such as RDF/XML (.rd) or JSON-LD (.json). All formats represent a knowledge graph.

As of now, the capture of provenance in health care is not adequately or uniformly implemented in German medical DICs, as revealed in a recent study on their data management status [23]. The results demonstrated that provenance is indeed a factor strongly influenced by the maturity level of data management practices. Following complex transformations in the data integration process, the provenance of data elements is often lost, making it difficult to impossible to assess the (measurement) quality of a data element. This reduction in traceability diminishes trust in the validity of the collected data.

The primary objective of this study is to improve the reusability of clinical routine data within a medical DIC for its secondary application in clinical research. Our goal is to enhance processed clinical routine data by incorporating appropriate semantic metadata, a key requirement guided by the FAIR principles [17]. Furthermore, our intention is to bolster the accountability of our DIC by mitigating the risks associated with the reuse of compromised data in clinical research.

To our knowledge, this is the first demonstration of provenance integration within a medical DIC.

## Methods

### Materials

We used test data to develop and test our provenance class. Test data elements were chosen to reflect the composition of a typical data integration repository. We created exemplary dummy data element definitions with comprehensive annotation (Textbox 2). We defined 7 data element types and generated 100,000 data elements for each data element type to generate a total of 700,000 provenance records using a Python (Python Foundation) script.

**Exemplary dummy text–based data element definition.**

id=’syst_blood_pressure’,

name=’syst_blood_pressure’,

description=’Systolic Blood Pressure’,

source=’stg_sap_vitalis’,

source_variable=’SysBP’,

destination=’dwh_vitalis’,

destination_variable=’SBP’,

description_of_transformation=’copy’,

description_of_qualitycheck=’range check 80-160’,

status_log=’passed date 12.May2022’,

sop_name=’SOP p’

sop_version=’v1.5’,

sop_status=’approved’,

steward_name=’no name given’)

### Proof-of-Concept Solution

**Figure 1:**
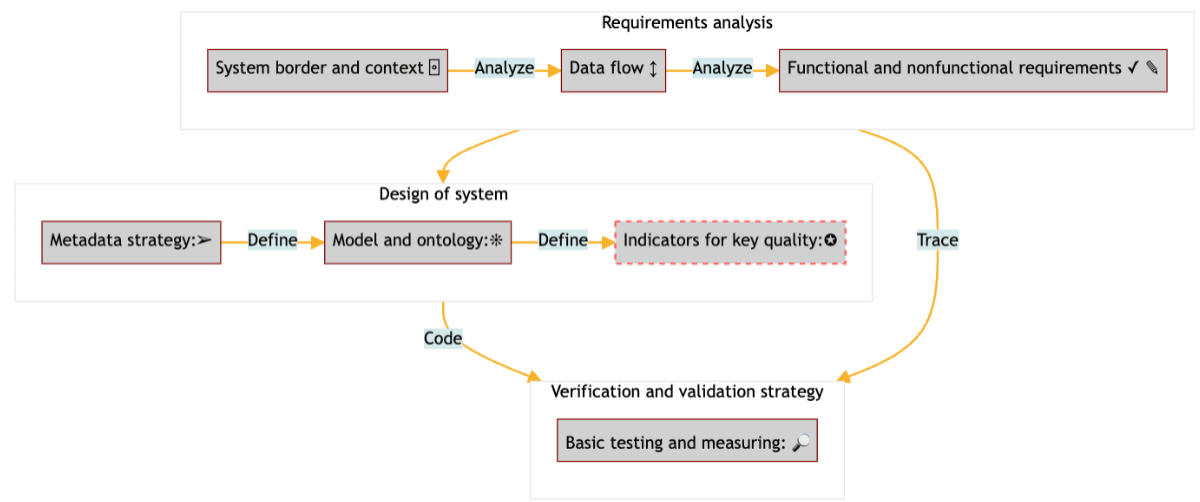
Overview of the road map steps.

Following the tailor-made provenance framework [3], we developed a proof-of-concept provenance solution. This framework complements a standard software engineering cycle (requirements, design, coding, testing, and implementation) with insights from a comprehensive literature search and uses established works as a guide to the users of the framework. The expanded requirements analysis is substantiated by the topics identified through the literature search. Details are described in Figure 1.

### Requirements Analysis

#### Overview

An interdisciplinary team of internal stakeholders in the University Medicine Mannheim-DIC (lead, medical experts, computer scientists, technical staff, and process owner of the ETL [extract, transform, and load] process) performed the requirements analysis for the research approach. Initially, we engaged in discussions, documented feedback, and obtained approval for our own data pipeline processes, based on the WH questions (what, when, where, who, why, how, which, whose). This was done to ensure accurate and risk-managed data processing pipelines. Our focus centered on questions related to data governance, annotation, documentation, interoperability, data integrity and accuracy, data sharing, and information technology operations. This emphasis aligns with a prior investigation on data management practices in German DICs [23], where these questions were identified as integral to tracing patient data through the DICs.

Building on the previous steps, we initiated the process by visualizing the scope definition (system border and context) of the planned provenance tracking systems. Using notation according to DeMarco [24], we generated a data flow diagram. Following this, we documented the resultant requirements, representing them in free text and as a unified modeling language (UML) class diagram to address various requirements perspectives [25].

#### System Border and Context

**Figure 2:**
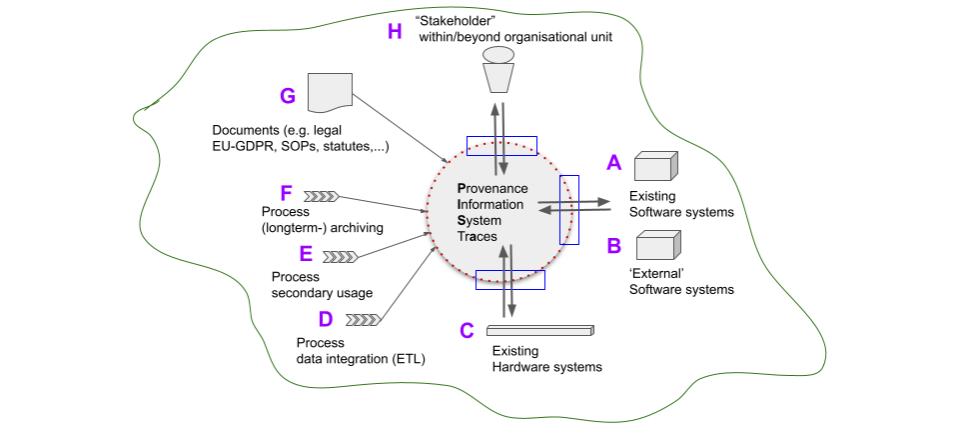
Aspects in the system context and border of the Provenance Information System Traces (PISA). EU: European Union; GDPR: General Data Protection Regulation; SOP: standard operating procedure.

The context view (Figure 2) is used to delineate the scope of our system, establishing the boundary between functionalities that are considered in and out of scope. The system to be modeled, known as the Provenance Information System Traces (PISA), is depicted as a circle in the center (outlined by the dotted red line in Figure 2). At the conceptual level, we established the system border to encompass all aspects within the object scope. We delineated the system context (depicted in green as a freehand drawing) with aspects (A to H) that impact the planned provenance tracking system in our medical DIC. The processes that were modeled had been previously defined by local stakeholders and were influenced by the processes of the medical informatics initiative community [5]. The core process, the ETL process (D), includes valid documents (G) (eg, statutes, standard operating procedures, European Union-GDPR) and the involvement of stakeholders within and beyond the organizational unit (H), representing the primary focus of our development efforts. Existing software and hardware systems (A–C), as well as the processes of secondary usage for data request (E) and long-term archiving (F), are outside the scope of this study.

#### Data Flow

**Figure 3:**
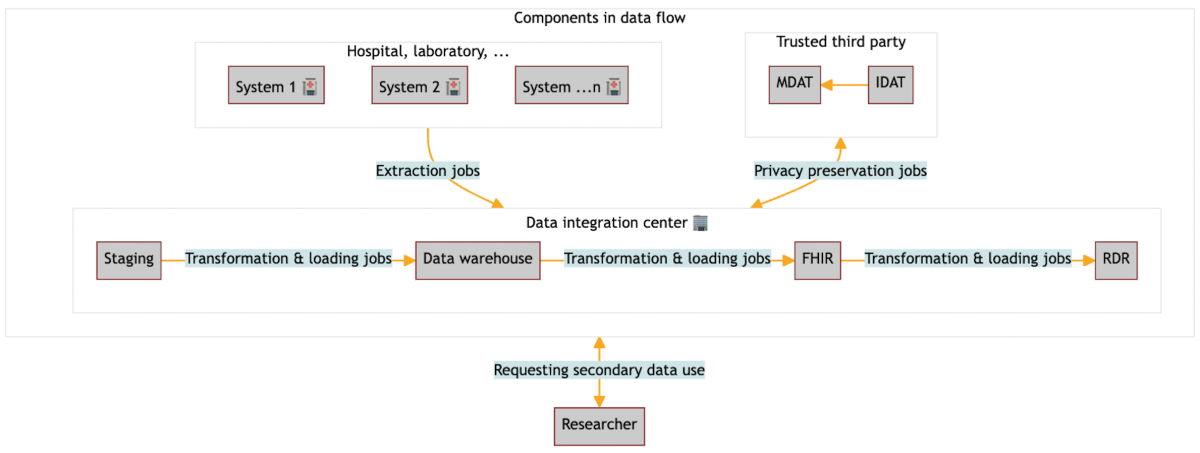
Simplified general data flow diagram in the data integration center. The simplified general data flow diagram in the data integration center (DIC) provides information about components participating in data flow: different hospital or laboratory systems donating the data, the independent trust center (trusted third party) enabling the separate processing of identifying data (IDAT) and medical data (MDAT), the data integration center with the different integration phases staging, data warehouse, FHIR and the research data repository (RDR). Individual DIC may deviate from this general data flow. FHIR: Fast Healthcare Interoperability Resources.

Given the multitude of processes within a DIC, we confined our focus to the requirements related to the data integration process (Figure 2; ETL, letter D). We scrutinized the data flow and derived a data flow diagram, illustrating the functional requirements perspective (Figure 3). As part of the Medical Informatics Initiative, all DICs in Germany modeled a comparable, generic data flow. This data flow delineates the movement of data among processes (ETL), storage entities (staging area, data warehouse, FHIR server, and research data repository), and involved actors (staff in DIC, researcher, and trusted third party). Processes encapsulate functions responsible for transforming processing data. These processes consume input data from diverse systems, manage these data, and convey the results to an output. Storage ensures data persistence, allowing processes to access the storage in read or write modes. Actors actively engage in information exchange with the system.

#### Requirements Description

In a previous publication, we conducted interviews with various German medical DICs [23]. Through these interviews, we identified the most crucial requirements, emphasizing assessments of data quality, traceability, and information capability. Additionally, transparency in processing steps, workflows, and data sets emerged as a significant consideration. Other identified requirements encompassed aspects such as debugging or performance evaluation. Additionally, there was a focus on compliance with regulations, reproducibility, support of the scientific utilization process, increased confidence in data, and clear regulation of responsible parties [23].

In alignment with this study, we established preconditions and requirements along the data flow for implementing the provenance tracking system. We identified the intended features for the implementation of the PISA and derived the system’s requirements (Table 1). In general, PISA should have the capability to trace the complete production history of a data element while incorporating domain-specific characteristics of the data element. These provenance traces for an individual data element must be captured along the presented data flow.

**Table 1:** Requirements for the proof of concept for PISA^a^.

Number	Requirements (functional and nonfunctional)	Explanation
1	PISA must have the capability to track the complete processing history of a data element, and the provenance information must be stored in a database. This encompasses all derivation steps performed on data elements during their processing steps.	It includes all the information (metadata) required for producing a specific data set or a data element while preserving its data integrity status. This encompasses details such as data source, data destination, method, tools, software, and versions used. The benchmark should align with the “entities” and “activities” components of the W3C model.
2	PISA must possess the capability to trace organizational responsibilities and the means used.	It includes information (metadata) about all the involved agents in producing a data set or data elements, such as staff, standard operating procedures, and guidance. The benchmark should align with the “agent” components of the W3C model.
3	PISA must be analyzable by an authorized user and capable of producing diverse representations and export formats for the provenance traces.	Detailed provenance traces are accessible and exportable to support evaluation by users, including formats such as log files, FHIR^b^ provenance, W3C^c^ RDF^d^/XML, and RDF/JSON-LD provenance.
4	PISA must be able to track the quality status and assessment of data elements.	The provenance information for a data element is expanded to include the quality status of the processed data element.
5	PISA must be able to track the status of the script execution.	At a minimum, the provenance information should encompass the verification status and time stamp of the processed scripts.
7	PISA must provide a high level of ease of use for ETL^e^ programmers and should be usable without requiring in-depth knowledge of provenance terms and concepts.	PISA should facilitate easy integration into ETL pipelines with transfer interfaces, allowing seamless integration with established technologies. Moreover, it must be easy to install, for example, by supporting widely used and easily set up databases.
8	PISA must be time-efficient and capable of ensuring acceptable performance.	Time measurements per data element must take place and be evaluated to verify the feasibility of the proof-of-concept approach.
9	Verification by unit tests/code coverage >80%	Passed testing results.

^a^PISA: Provenance Information System Traces.

^b^FHIR: Fast Healthcare Interoperability Resources.

^c^W3C: Word Wide Web Consortium.

^d^RDF: Resource Description Framework.

^e^ETL: extract, transform, and load.

### Design and Architecture of the Provenance Class

#### Development of the Logical Data Model

**Figure 4:**
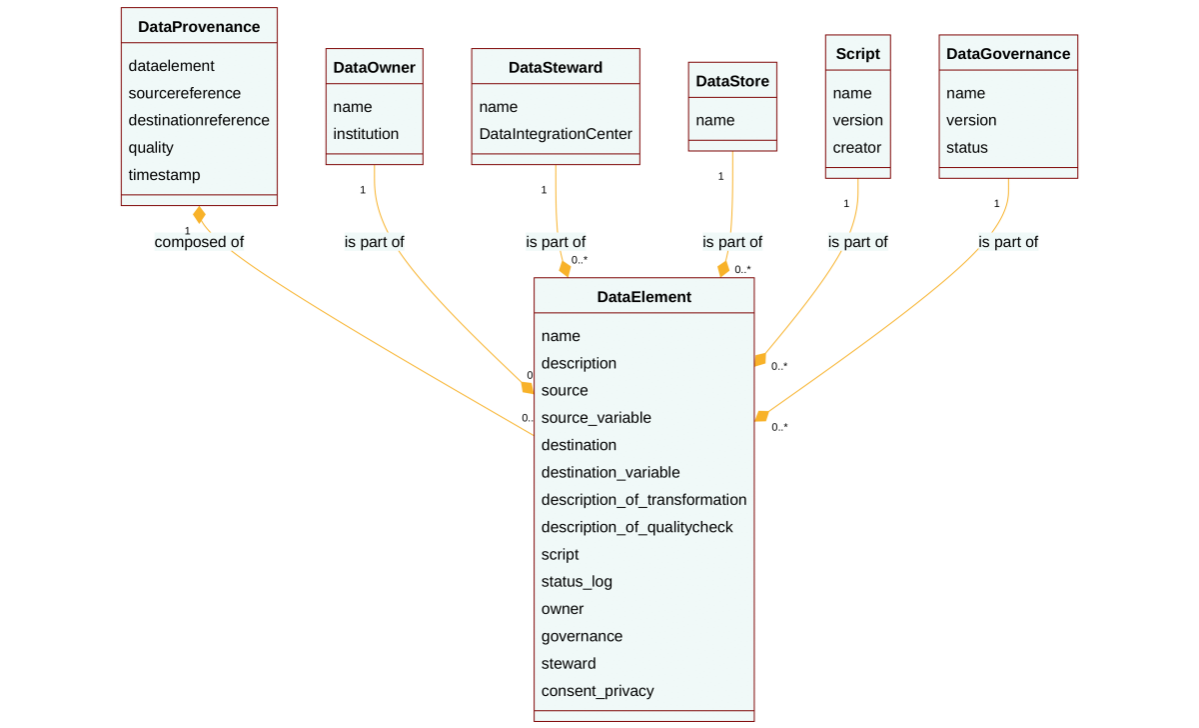
The logical data model as UML class diagram (technology-agnostic).

Based on the aforementioned requirements (Table 1) and the DIC maturity model [23], we constructed the logical data model as a UML class diagram, identifying classes and their associations (Figure 4).

#### Metadata Strategy

Our metadata strategy centered on characterizing the data elements and their associated artifacts throughout their processing pipeline.

Aligned with the requirements and the logical model, we extracted the pertinent provenance metadata and aligned this provenance profile with the W3C components entity, agent, and activity. Simultaneously, we diligently enforced documentation efforts and annotation, guided by good documentation practices such as the ALCOA(+) principles for the identified components [10]. The annotation process we implemented enhanced the comprehension, increased understanding, and improved the traceability of the processed data elements.

The FAIR principles R1.2 and R1.3 guided us to enrich (R) data elements with meaningful (provenance) metadata. Consequently, we characterized data elements by collecting content-rich contextual and technical metadata that narrate the story of the entire data processing workflow and link to related artifacts (Table 2). During the transformation processes, we documented quality procedures and incorporated coding practices and versioning information.

**Table 2:** Levels of contextual and technical metadata and their related FHIR^a^ mapping: a mapping example of our metadata to the FHIR Provenance resource. The FHIR Provenance elements are aligned with the W3C^b^ PROV model elements.

Level^c^	Description^d^	Possible mapping^e^	Exemplified output^f^
Data Governance^g^	Name and version of the standard operating procedures or regulation (eg, “DIC_ETL-ST.pdf, v1, approved”)	.policy .agent.type	“policy” : [“http://example.org/policy/1234”], “location”: { “reference”: “DIC” },
Data Owner	Name of the (hospital) department and the responsible person owning the patient data (eg, physician or stakeholder name)	.authorization .agent .agent.type .agent.role .agent.who .agent.onBehalfOf	“authorization”: { “coding”: [ { “system”: “http://terminology.hl7.org/CodeSystem/v3-ActReason”, “code”: “TRANSRCH” } ] },
Data Steward	Name of the responsible data steward (eg, person who takes care of data management)	.location .agent .agent.type .agent.role .agent.who .agent.onBehalfOf	“agent”: { “who”: { “display”: “Hr. Koch” } }
Data Store	Used input or created output data file as part of the processing pipeline (eg, name original source system and name target system)	.entity .entity.role .entity.what .target (as mapping from entity)	“entity”: { “what”: { “identifier”: [ { “system”: “urn:ietf:rfc:3986”, “value”: “243c773b-8936-407e-9c23-270d0ea49cc4”, “display”: “” } ] } }
Data Script	Scripts or programs developed to process the data with a description of script version and name and creator (eg, etl_st.py v1 MZ)	.activity .basedOn .agent.type	“activity”: { “coding”: [ { “system”: “http://terminology.hl7.org/CodeSystem/iso-21089-lifecycle”, “code”: “averaging”, “display”: “Transform” } ] } “basedOn”: [ { “reference” : “ServiceRequest” } ]
Data Element	Individual characteristics per data element during a processing step such as ID, name, description, source and destination information from Data Store Level, description of the transformation approach, description of quality check (testing and validation approach), privacy and security status, and information from Script Level	.entitiy .entity.role .entity.what .entity.agent	Schema as in Data Store Level
Data Provenance	References to all other mentioned levels and testimony for quality (eg, “25, 3, 5, good, 2023-02-03 06:01:34”)	.id .occuredDateTime .recorded .patient .encounter .target	“id” : “id” ”occuredDateTime“: ”timestamp“, ”recorded“: ”timestamp“
Data Infrastructure^g^	Used hardware and software conditions during data processing	N/A^h^	N/A

^a^FHIR: Fast Healthcare Interoperability Resources.

^b^W3C: Word Wide Web Consortium.

^c^Level corresponds to the maturity level of the data integration center.

^d^Description of the possible content or annotation.

^e^Possible mapping to the Health Level 7 FHIR resource “Provenance.”

^f^One possible exemplified output extract as a serialization in FHIR JSON.

^g^Not yet or only partly implemented.

^h^N/A: not applicable.

Examples of expanded metadata elements are more detailed descriptions of the transformation, the quality check, and the status of the data element in scope, or the results of the used log files. The metadata gathering for provenance comprises both manual annotation and an automated collection process, representing a hybrid form of provenance [26].

#### Ontology

**Figure 5:**
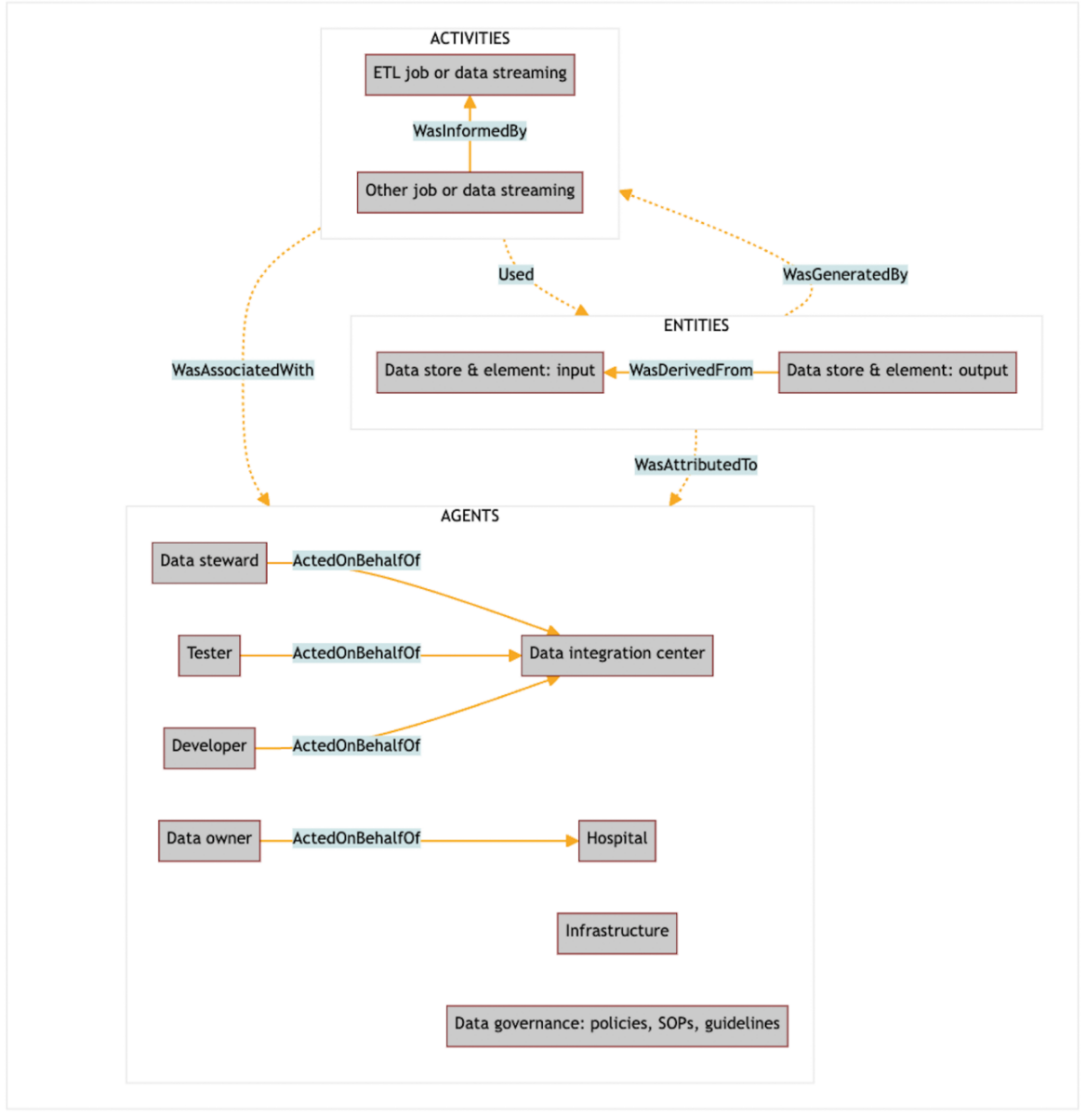
Exemplary instantiation of the provenance information model. SOP: standard operating procedure.

We organized, annotated, and represented information using WebProtégé 4.0.2 (Protege Team in the Biomedical Informatics Research Group at Stanford University), a tool designed for collaboratively creating complex ontologies [27]. The W3C PROV ontology and the fundamental relationships between entities, activities, and agents served as a framework for representing the provenance graph [20]. More specifically, we mapped processes onto activities, actors onto agents, and input/output data onto entities. The attributes of the provenance data model were aligned with the attributes of the data set. An instantiation of the provenance model, reflecting the W3C PROV vocabulary and layout convention, is illustrated in Figure 5. Additionally, the W3C PROV supports interoperable interchange of provenance in heterogeneous environments.

#### Implementation and Verification Approach

Finally, building on the preceding steps, we developed an open-source Python class “Data Provenance” with associated methods, and validated our approach in an exemplified data integration pipeline [28]. Provenance traces were mapped exemplarily onto the W3C RDF/XML and HL7 FHIR resource “Provenance” in its current maturity level (version R 5). We utilized peewee (version 3.15.4), a Python Object-Relational Mapping library that supports the binding of objects to relational databases such as SQLite, MySQL, or PostgreSQL [29]. To visualize the provenance traces, we used the Mermaid plotting framework [30].

The verification and validation approach for the developed provenance class involved an independent code review and unit tests to ensure that the code meets the requirements of the design. We assessed efficiency (storage space in kilobytes and computing time) and ensured the maintainability of the program (code structure, modularity, comments in code, currency, and comprehensibility of documentation).

While creating provenance records, we conducted a runtime experiment to measure the performance of our developed class. We recorded the time that the program took to run for proper execution. The runtime environment comprised the operating system Ubuntu 22.04.2 LTS (Canonical Ltd.), 32 GB memory, and an 8-core Intel Xeon Platinum 8276 CPU @ 2.20-GHz computer.

As a runtime environment, we used a virtual machine running on top of the machine. The runtime period was defined as the duration when the program was actively running.

We conducted measurements per data element and per provenance record on 9 virtual machines, each utilizing different data element block sizes (starting with 1, 10, 100, 1000, 10,000, and 100,000 up to 9, 90, 900, 9000, 90,000, and 900,000 data elements). For the analysis of runtime measurements, we used R version 4.2.0 (2022; R Foundation for Statistical Computing), and figures were generated using the ggplot2 package [31].

The code is available in a git repository under the Massachusetts Institute of Technology (MIT) license [32].

### Ethical Considerations

Given the nature of the proof-of-concept study relying on dummy test data, ethics approval, informed consent, and deidentification were not applicable.

## Results

### Provenance Traces Representation

All the gathered provenance information is in a machine-readable format. Additionally, FHIR health care standards were used [33].

We developed an FHIR profile based on the “provenance” resource, resulting in a record that delineates the entities and processes involved in producing, delivering, or otherwise influencing that resource. This was accomplished by mapping the contextual and technical metadata to the corresponding resource provenance elements (Table 2).

Through the integration of all metadata levels, we facilitated the traceability of each data element. We illustrated the traceability using a data flow diagram and presented it in a human-readable text form. Additionally, the provenance information was exported into various formats such as FHIR-JSON, W3C-RDF/XML, W3C-RDF/JSON-LD, or a text-based log file. This approach aligns with data obtained in other studies [34].

### Measurement of Provenance Traces

**Figure 6:**
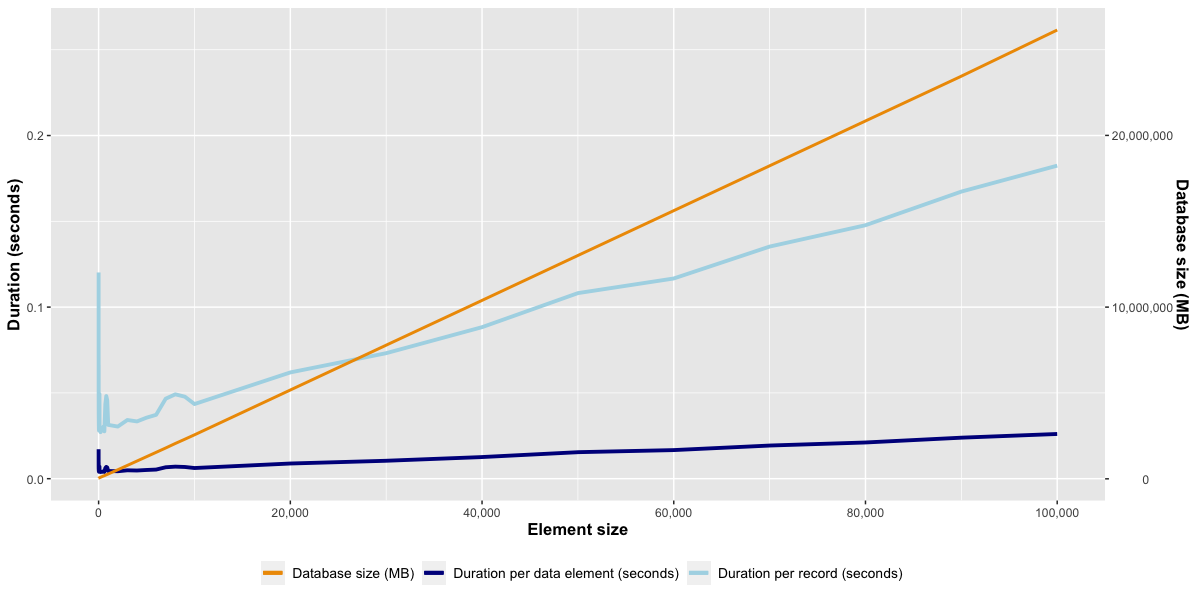
Provenance-Runtime-Experiment presenting storage duration per element and per record.

As anticipated, the specified provenance class successfully generated the database and the metadata tables according to the UML class diagram (illustrated in Table 2). Provenance records were automatically appended to the provenance table throughout the execution of the exemplified data integration pipeline. We recorded runtime measurements of the algorithm, displayed separately for the storage duration of a data element and for a record, as well as the corresponding increase in the database (Figure 6). As evident, the runtime complexity of the algorithm per data element indicates a nearly linear relationship with the size of the input data.

We observed an acceptable runtime duration ranging from 0.0039 to 0.02601 seconds per data element. However, when measuring the runtime for a provenance record, we encountered an increasing duration, ranging from 0.0271 to 0.1882 seconds. Given that our approach incorporates novel aspects, we were unable to find comparable studies for this measurement. Nevertheless, the data obtained here suggest that using this approach to establish provenance traces can yield accurate and timely information.

### Verification and Validation

The validation status for our proof-of-concept provenance class is outlined in Table 3. We anticipate that our results can be readily adopted for additional metadata components and seamlessly transferred to decision-making applications.

**Table 3:** Validation status of requirements.

Requirement number	Validation result
1	Introduction of metadata for data elements and their processing collected automatically during ETL^a^ job running in data flow. Relevant tables (DataProvenance, DataElement, and associated tables) in the provenance database were created and continuously updated during processing.
2	Organizational topics (DataGovernance, DataSteward, and DataOwner) were recorded in the provenance database and continuously updated during processing.
3	Provenance traces were created in different formats. Detailed provenance traces are accessible and exportable to support evaluation by users (eg, FHIR^b^ provenance, W3C^c^ RDF^d^/XML RDF/JSON-LD provenance).
4	The quality status of a processed data element is tracked and currently presented with a placeholder value in the DataProvenance table (see the “Future Work” section).
5	The verification status of used scripts and time stamps were recorded in the table DataElement. More specific content-related provenance information needs to be added in the second step. This compromises detailed annotation about the performed transactions and can be used for handling inconsistencies and rules for conflict resolution (see the “Future Work” section)
7	Easy integration into the ETL pipeline setup: only 3 lines of code, set up per data element: 1 line (see the “Future Work” section).
8	Time measurements confirmed satisfying results.
9	We achieved a code coverage of >90%, confirming that the code is comprehensively verified (quality aspect for software). We successfully verified the provenance with unit tests and validated all results against the defined requirements.

^a^ETL: extract, transform, and load.

^b^FHIR: Fast Healthcare Interoperability Resources.

^c^W3C: Word Wide Web Consortium.

^d^RDF: Resource Description Framework.

## Discussion

### Principal Findings

Our study introduces the first ready-to-use library designed to record provenance information from clinical data processing pipelines in a German medical DIC. This current research extends previous work in provenance by using an approach that systematically combines detailed insights from medical, data management, and information technology operational experts. This method aims to facilitate the reuse of enriched patient data with precision and rigor. We demonstrated that our research approach successfully facilitates the implementation of traceability in the processing of data elements. This, in turn, contributes to the promotion of good data management and documentation practices, ultimately ensuring sufficient provenance quality. Furthermore, these good practices pave the way for the (automated) generation of annotations [23] and prevent poor data integrity, thereby enhancing data quality [35]. Through this, we hypothesize that our work could contribute to the reliability and safety of quality-assured patient data for secondary use. Simultaneously, we mitigate the risks associated with the reuse of weak data in clinical research.

We fulfilled the requirement for FAIR (Findability, Accessibility, Interoperability, and Reusability) provenance information by adhering to standards for syntactic and semantic interoperability, including JSON, W3C PROV, and FHIR mapping. Compared with the FHIR resource Provenance, we noted that our metadata recording offers significantly more detailed contextual information for each data element. We suggest that improvements to the FHIR Provenance resource, particularly for data within medical DICs, be deliberated and harmonized with existing FHIR resources such as “AuditEvent” or the “FiveWs Pattern” [19].

The strengths of this study are (1) the provision of provenance information for data elements with export options to interchange standard formats such as FHIR-JSON or W3C RDF/XML; (2) the simplicity of integrating this provenance class into ETL and other data pipelines; and (3) the extensibility of metadata components along with acceptable runtime measurements.

### Related Work

In general, research on provenance and related management has progressed significantly in recent years. Numerous studies have been conducted, both domain specific and domain independent, focusing on provenance. Recently submitted scoping review results on provenance tracking have yielded valuable insights and provided an extensive summary of current approaches and criteria [3]. The scoping review revealed technical, implementation, and knowledge gaps, with a specific emphasis on modeling and metadata frameworks for (sensitive) scientific biomedical data. Moreover, the primary focus of the research was centered on workflow provenance. This involved the utilization of models such as the Open Provenance Model or the W3C PROV data model across various semantic levels and tools in scientific workflows or experiments, as demonstrated in frameworks such as BioWorkbench or the OpenPREDICT use case [36,37]. Additionally, other work has delved into different yet more general approaches for metadata usage and harvesting [38,39]. A systematic literature analysis on functional requirements for medical data integration outlined general requirements for data traceability and metadata management [40].

While these prior efforts are crucial, they still lack the specific requirements and considerations tailored for a DIC use case. By contrast, our approach is finely tuned to the unique needs of a DIC, providing a comprehensive exploration of provenance that imparts medical meaning and understanding to the data elements, thereby enhancing their reusability.

### Lessons Learned

We discovered that interdisciplinary competence profiles; fostering communication between medical experts, data stewards, and information technology developers; and establishing a common language were pivotal factors leading to significant progress in our specific DIC use case. Implementing proper data governance and comprehensive data management documentation, such as data management plans, would be instrumental in mitigating the risk of incorrect use of the data.

The lessons learned from our description could serve as motivation for other researchers aiming to establish FAIR-oriented provenance. This would not only advance the reuse of their research data and results but also underscore the importance of maintaining overall responsibility for the data, even after project funding concludes.

### Future Work

Future work should also prioritize the development of a strategy for assessing data privacy, data integrity, and related quality of a data element. Integrating this information into the framework would enhance the expressiveness of the provenance information and enable the derivation of quality dimensions. For this reason, data elements may need to be accompanied by additional properties (refer to Table 2) that are significant for interpretability, helping determine limitations or detect duplications for use in similar research studies. Addressing the adequacy and relevance of the data element for upcoming research questions aids in supporting interpretation and, consequently, the reuse of a data element, as already highlighted in a draft Food and Drug Administration guidance [41]. To facilitate easy integration with other programming languages, we will provide an application programming interface.

Future studies should also explore ways to enhance the script for generating the provenance class in alignment with the FAIR for Research Software Principles [42]. Determining appropriate software metadata that accurately describe the specific characteristics of the software is an essential aspect to be addressed [18].

Before the future implementation and integration of the provenance class into real-world data integration processes, it is advisable to seek recommendations for risk measures. Factors such as the confidentiality level and security of provenance information, storage considerations, performance issues, and scalability should be carefully considered. In addition, it is crucial to consider experiences gained from maintaining metadata management and interoperable technologies, especially from professional data stewards. Ongoing exchanges with stakeholders and conducting usability evaluations are essential aspects that should be taken into account.

This work also contributes to a broader community project that seeks to establish the “Minimal Requirements for Automated Provenance Information Enrichment” (MIRAPIE) project [43].

### Limitations

As the library has only been tested with simulated data, the next step—testing in a real environment—is currently in preparation. Despite the straightforward ETL integration approach, we will carefully assess the complexity and associated costs of implementation within the medical DIC. We recognize the need to bolster the overall qualification and validation concept. We believe it is crucial to expand the current provenance class to one that is inspection- or audit-ready, although accreditation demands additional measures and efforts. Additionally, further scalability analysis should be incorporated into the research approach.

Trust involves more than just the provenance of data elements; it also implies correctness and security against malicious users. This challenge can only be addressed through technical access limitations and organizational measures. Nevertheless, automated provenance traces can contribute to building trust in the transformation and movement of data within the DIC. Moreover, it empowers us to confidently assess the quality and validity of the original data points even after undergoing complex transformations within a data warehouse.

### Conclusions

We have designed, developed, and implemented provenance traces at the data element level for a German medical DIC, with the potential for extension at the national level. The described research method for the proof-of-concept provenance class has been crafted to promote effective and reliable core data management practices, enriching biomedical data with meaningful provenance. This, in turn, strengthens the benefits for research and society while simplifying the reuse of biomedical data. While the approach was initially developed for the medical DIC use case, these principles can be applied universally throughout the scientific domain. The implementation and analysis of provenance traces play a crucial role in minimizing risks associated with undetected or unintended data integrity breaches. Hence, provenance traces significantly contribute to building trust in routine clinical data and enhancing the accountability of a medical DIC. We are confident that by adhering to this advanced practice, the existing gaps between industry (pharmaceutical companies), service providers, and academia can be mitigated. Consequently, this can lead to an increase in the secondary use of (sensitive) patient data in clinical investigations.

The outcomes of our research prompt additional questions, particularly regarding how in-depth exploration of further provenance analysis can predict the quality of data using machine learning methods. The limitations identified in our study indicate the need for further investigations into provenance theory, standards, and practices in the clinical field.

## Abbreviations

ALCOA: Attributable, Legible, Contemporaneous, Original, and Accurate
DIC: data integration center
ETL: extract, transform, and load
FAIR: Findability, Accessibility, Interoperability, and Reusability
FHIR: Fast Healthcare Interoperability Resources
GDPR: General Data Protection Regulation
MIRAPIE: Minimal Requirements for Automated Provenance Information Enrichment
MIT: Massachusetts Institute of Technology
PISA: Provenance Information System Traces
RDF: Resource Description Framework
UML: unified modeling language
W3C: Word Wide Web Consortium

## Data Availability

The code of the provenance class is provided in a git repository [32].
